# A Novel Diagnostic Decision Support System for Medical Professionals: Prospective Feasibility Study

**DOI:** 10.2196/29943

**Published:** 2022-03-24

**Authors:** Joanna Timiliotis, Bibiana Blümke, Peter Daniel Serfözö, Stephen Gilbert, Marta Ondrésik, Ewelina Türk, Martin Christian Hirsch, Jens Eckstein

**Affiliations:** 1 CMIO Research Group Digitalization & ICT Department University Hospital Basel Basel Switzerland; 2 Ada Health GmbH Berlin Germany; 3 Else Kröner Fresenius Center for Digital Health University Hospital Carl Gustav Carus Dresden Technische Universität Dresden Dresden Germany; 4 Institute for Artificial Intelligence in Medicine Philipps University of Marburg Marburg Germany; 5 Department of Internal Medicine University Hospital Basel Basel Switzerland

**Keywords:** diagnostic decision support system, DDSS, probabilistic reasoning, artificial intelligence, dyspnea, emergency department, internal medicine, symptom checker

## Abstract

**Background:**

Continuously growing medical knowledge and the increasing amount of data make it difficult for medical professionals to keep track of all new information and to place it in the context of existing information. A variety of digital technologies and artificial intelligence–based methods are currently available as persuasive tools to empower physicians in clinical decision-making and improve health care quality. A novel diagnostic decision support system (DDSS) prototype developed by Ada Health GmbH with a focus on traceability, transparency, and usability will be examined more closely in this study.

**Objective:**

The aim of this study is to test the feasibility and functionality of a novel DDSS prototype, exploring its potential and performance in identifying the underlying cause of acute dyspnea in patients at the University Hospital Basel.

**Methods:**

A prospective, observational feasibility study was conducted at the emergency department (ED) and internal medicine ward of the University Hospital Basel, Switzerland. A convenience sample of 20 adult patients admitted to the ED with dyspnea as the chief complaint and a high probability of inpatient admission was selected. A study physician followed the patients admitted to the ED throughout the hospitalization without interfering with the routine clinical work. Routinely collected health-related personal data from these patients were entered into the DDSS prototype. The DDSS prototype’s resulting disease probability list was compared with the gold-standard main diagnosis provided by the treating physician.

**Results:**

The DDSS presented information with high clarity and had a user-friendly, novel, and transparent interface. The DDSS prototype was not perfectly suited for the ED as case entry was time-consuming (1.5-2 hours per case). It provided accurate decision support in the clinical inpatient setting (average of cases in which the correct diagnosis was the first diagnosis listed: 6/20, 30%, SD 2.10%; average of cases in which the correct diagnosis was listed as one of the top 3: 11/20, 55%, SD 2.39%; average of cases in which the correct diagnosis was listed as one of the top 5: 14/20, 70%, SD 2.26%) in patients with dyspnea as the main presenting complaint.

**Conclusions:**

The study of the feasibility and functionality of the tool was successful, with some limitations. Used in the right place, the DDSS has the potential to support physicians in their decision-making process by showing new pathways and unintentionally ignored diagnoses. The DDSS prototype had some limitations regarding the process of data input, diagnostic accuracy, and completeness of the integrated medical knowledge. The results of this study provide a basis for the tool’s further development. In addition, future studies should be conducted with the aim to overcome the current limitations of the tool and study design.

**Trial Registration:**

ClinicalTrials.gov NCT04827342; https://clinicaltrials.gov/ct2/show/NCT04827342

## Introduction

### Background

Digital tools play an increasingly relevant role in the health sector. Most patients search the internet to complement their knowledge of health care topics [1]. In addition, patients increasingly use *symptom checkers* instead of standard search engines for symptom analysis [2]. In contrast to the fast uptake in the consumer sector, professional tools similar to symptom checkers designed to support physician decision-making have not found widespread adoption in the clinical and outpatient environment [3] even though this concept is not new. A variety of digital technologies and artificial intelligence–based methods are currently available and have recently emerged as impressively persuasive tools to empower physicians in clinical decision-making and improve health care quality [4]. Diagnostic decision support systems (DDSSs) have been demonstrated to facilitate the assessment of clinical data input by using an extensive medical knowledge base [5,6].

Continuously growing medical knowledge and the increasing amount of data make it difficult for medical professionals to keep track of all new information and to place it in the context of existing information [7]. DDSSs have been suggested as a solution to this problem [8]. An expert system can help by expanding the clinician’s differential diagnosis list and suggesting other avenues of investigation [9].

Diagnostic errors, consisting of inaccurate, delayed, or missed diagnoses, remain major challenges in public health care [10] that need to be addressed.

The overall purpose is to invite physicians to rethink and re-examine their steps and possible alternatives in light of the presented diagnostic information [11]. DDSSs are not intended to replace physicians but rather to augment and optimize the diagnostic decision-making process. If they are to be adopted, it is important that they provide accurate information and are trusted by clinicians. The diagnostic decision-making process must be as transparent and comprehensible as possible. The trustworthiness of the data handling and the medical quality of the knowledge base and algorithms are essential to this. Poor usability is another important barrier that could limit adoption and be a deterrent to the routine use of new technology.

In this study, we pilot-tested whether the use of a DDSS prototype from Ada Health GmbH is feasible in an emergency department (ED) setting.

### The Diagnostic Decision Support Tool

The DDSS is a web-based diagnostic decision support system for medical professionals developed as a research prototype by Ada Health GmbH that can be accessed by laptop or tablet.

In the DDSS, the physician can input a patient case over time with several visits (if relevant), and the system updates the provided decision support dynamically. The user interface consists of pages representing the steps during an individual patient visit and a case overview page. The design of this prototype provides full transparency over the artificial intelligence–based medical reasoning. The user interface allows for a continuous and transparent exchange between the machine and human.

The case starts with the input of the epidemiological data followed by a consultation page where one or several findings are entered. On the case analysis dashboard, symptoms, findings, and their related attributes can be added as *present* or *absent* for the case. The search allows the user to enter synonyms or related terms to find a specific symptom. In addition, the tool suggests a ranked list that changes in real time of symptoms and findings that have the highest potential for information gain for the current case. The DDSS supports the collection of both patient-reported complaints and findings gathered via medical examination or testing. Lifestyle or risk factors that may affect the patient’s condition can also be recorded, and it is taken into consideration via the reasoning engine. The system does not use a predefined standard ontology or taxonomy to enter symptoms.

**Figure 1:**
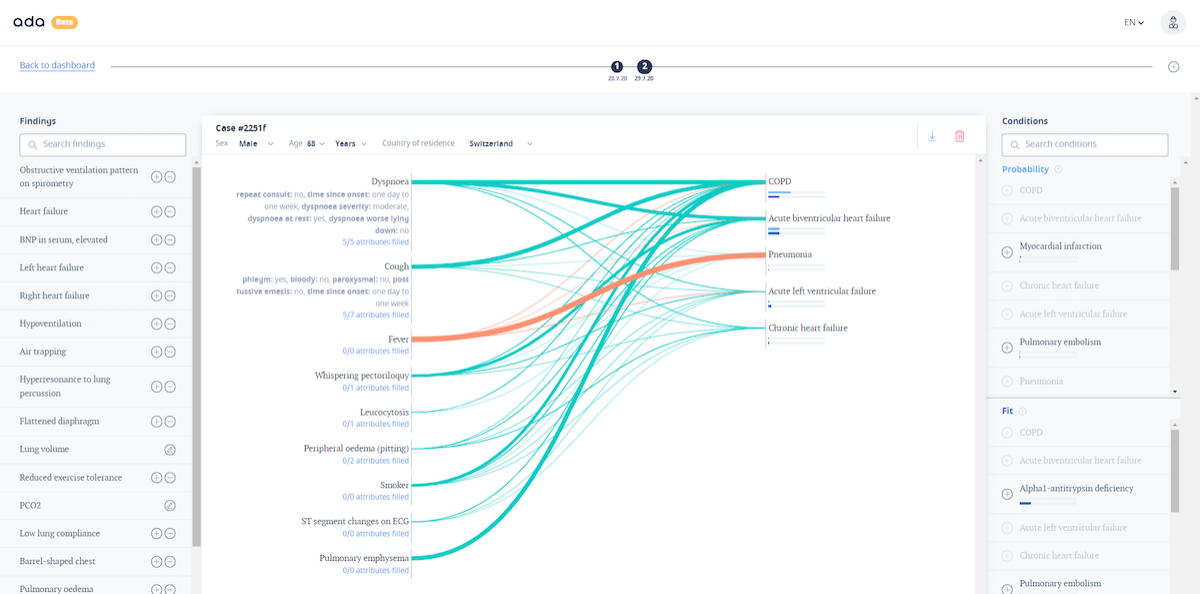
Screenshot of the case analysis dashboard of the diagnostic decision support system prototype.

The patient information, symptoms, and findings, as well as a list of differential diagnoses ranked by *probability* and *fit*, are represented on the main page of the tool (Figure 1).

The *probability* list is ranked by the estimated probability of a disease. It is based on the representation of medical knowledge using a probabilistic reasoning engine considering existing epidemiological data such as age, sex, or geographical location. This mirrors the approach that a health care professional takes during clinical routine. The *fit* list is ranked by the most likely conditions that could explain the finding constellation without knowledge of the probability of the conditions occurring in the general population. The reasoning engine infers disease probability estimations based on a representation of medical knowledge. The medical knowledge base is used to define a Bayesian network in which approximate inference is carried out.

Contribution lines visualize the correspondence between a symptom and a disease. The relative weighting of the symptoms to the diseases is indicated by the thickness of the lines. The color of the lines indicates the presence or absence of the finding in the constellation. This user interface was designed to ensure the transparency of the underlying reasoning engine inferences to the physician in real time. The medical knowledge base of the prototype DDSS was not based on a pre-existing database or medical knowledge ontology. Instead, it was generated and reviewed by in-house medical professionals using a process of curated integration of peer-reviewed medical literature. The medical knowledge and reasoning of the tool were designed with the primary goal of achieving high condition suggestion accuracy. More than 1300 conditions and 11,000 findings and symptoms are available in the medical knowledge base.

The DDSS prototype has been examined in a retrospective study with a focus on rare diseases, demonstrating that Ada suggested accurate diagnoses earlier than clinical diagnoses in more than half of all cases [12]. However, this tool has not been investigated prospectively in a real-life setting. The DDSS is a prototype in development, has not yet been optimized for everyday use, and is not publicly available. Nevertheless, the user interface is novel and unique in its presentation and transparency. In this regard, we aimed to conduct feasibility testing with a focus on a very common symptom; namely, *dyspnea*. Patients presenting to the ED with dyspnea were chosen as the focus area for testing as dyspnea has a wide range of possible etiologies, including cardiac, pulmonary, and infectious diseases [13]. This approach ensured a broad range of possible outcomes to comprehensively test the system’s novel user interface while being appropriate to the stage of development of the prototype.

### Aim of the Study

This is the first prospective study evaluating a DDSS prototype from Ada Health GmbH, which uses a novel approach for dynamically interacting with the physician in a real-life clinical setting by entering routinely collected health-related personal data. Our primary goal is to investigate the potential of this concept. Secondary outcomes are the identification of any key reasons for inaccuracy, current technical limitations, and the potential for further development and adaptation of the DDSS prototype based on the findings and needs identified with regard to the usability of the tool.

## Methods

### Study Design and Case Selection

We conducted this prospective feasibility study (ClinicalTrials.gov: NCT0482734) at the ED and internal medicine ward of the University Hospital Basel, Switzerland. A convenience sample of 20 adult patients admitted to the ED with a chief complaint of dyspnea and a high likelihood of inpatient admission was selected. The participants had to be able to understand, speak, and read in German. The exclusion criteria were refusal of consent and discharge from the ED without inpatient admission. The study period was from May 2020 to August 2020. The study participation of each patient lasted as long as the patient stayed in the hospital.

**Figure 2:**
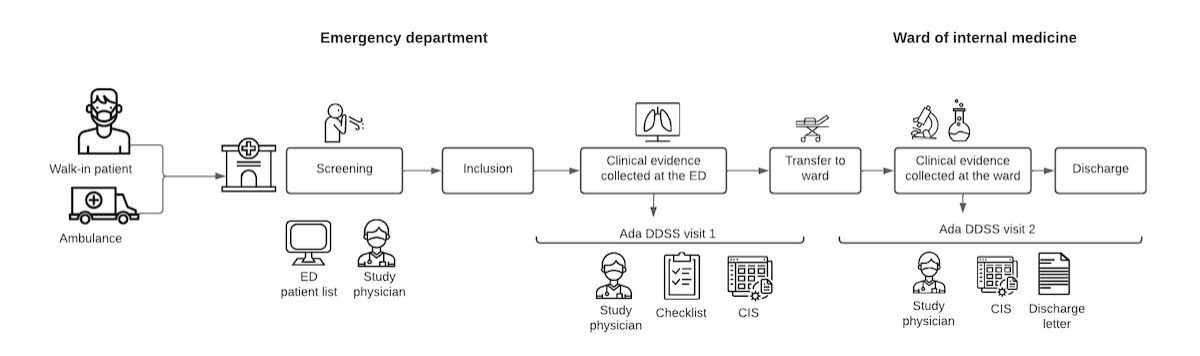
Study process. CIS: clinical information system; DDSS: diagnostic decision support system; ED: emergency department.

This study design was observational—patients with dyspnea admitted to the ED of the University Hospital Basel were monitored, diagnosed, and treated according to the usual clinical routine. The study physician (PDS) shadowed the treating physician and the patient throughout the entire hospitalization without any interference with the routine clinical work (Figure 2).

A first evaluation of potential patients for recruitment against the inclusion and exclusion criteria was based on a patient’s medical file using the triage findings from the ED. The decision whether the patient could be included was made after the first patient contact (ie, within hours of the patient’s arrival at the ED). The investigator explained the objective of the study and its observational nature to the patient. For ethical and organizational reasons, consent from the patient was obtained post hoc once the patient was hospitalized (following Human Research Act Article 31 [14] and Clinical Trials Ordinance Article 15 [15]). Data from patients who refused to provide post hoc consent were no longer used for the research project.

### Data Acquisition

#### Data Collection

All patients underwent an initial clinical assessment at the ED in which the study physician used a checklist to document symptoms, medical history, vital signs, and physical examination in a structured manner. Complementary information documented by the treating care team during hospitalization as well as all other investigation findings were extracted from the medical record.

The treating physician at no point had access to insights into the case from the DDSS prototype. The patients received usual care from their examining and treating medical staff. The study investigator (PDS) was not involved in patient care at any point.

#### Prototype DDSS Input

Once a patient was admitted, a new case was created in the DDSS with the patient’s sex, age, and geographical location as the first information. As we focused on patients with dyspnea in this study, a new patient case was started by entering the finding *Dyspnea* and selecting the corresponding attributes and specifications. All clinical evidence collected from the patient was entered as DDSS input data to build the case (Figure 1). Findings that would have been marked as *absent* (eg, no fever) were only added if relevant to the list of diagnoses or if explicitly mentioned in the medical record.

Information from the medical record was assigned to the time of the visit in the DDSS prototype. The idea was to mirror the patient’s journey in the hospital and provide the system with the same amount of information the treating physician had at a certain point in time. The first visit (visit 1) in the DDSS was created at the end of the ED stay. All evidence prospectively collected until this moment was entered into the DDSS. The second visit (visit 2) in the DDSS corresponded to when the patient was discharged from the hospital. All information from the first visit was transferred to the second visit, modified if necessary, and complemented with additional information from the patient file gained during the hospital stay. Any information of potential relevance to diagnosis that could not be entered into the DDSS as it was not found in the tool was recorded in a separate document. Missing diagnoses were also noted.

An additional visit (visit 2.1) in the Ada DDSS was performed retrospectively by a physician and former Ada employee with expert knowledge of the medical content and technical aspects of the Ada DDSS. This person screened the clinical cases and lists of missing information in the tool generated by the study research team. The goal was to show the user dependency of the DDSS and the influence of this on the accuracy of the DDSS suggestions.

All inputs were performed in German as all clinical evidence was gathered in German.

### Feasibility and Usability of the DDSS

The time of data entry, search functions and functionalities, availability of findings and diagnoses, and applicability of the tool in an acute ED setting were recorded to assess the feasibility of the DDSS prototype. We also evaluated the workflow and whether the navigation, data entry, and retrieval would impede clinical task completion. Furthermore, the input procedure with the tool’s robustness to irrelevant variations in input data as well as the technical aspects and potential restrictions were analyzed.

The usability was assessed by considering the structure and composition of the DDSS interface and whether it was satisfactory.

As the novelty of the tool is mainly reflected in the design of the interface, this was a key object of investigation. Therefore, the clarity of the visual representation of clinical data and the ease of acquiring information at a glance were examined in detail.

The guidance through different levels of the tool (onboarding, consultation page, and case analysis) was another point of interest. We tested whether the logic and availability of the desired options were consistent and rigorous and if the tool provided an effective layout. A risk evaluation of the misinterpretation of information was conducted.

### Metrics

Different metrics for the assessment of the accuracy of the DDSS suggestions are listed and defined in Textbox 1.

**Metrics for the assessment of the accuracy of the diagnostic decision support system suggestions [5].**

- Correct or accurate diagnosis retrieval: proportion of cases in which the correct diagnosis was the first diagnosis listed (M1), listed as one of the top 3 (M3), or listed as one of the top 5 (M5)
- Diagnosis in knowledge base: proportion of the diagnoses that were included in the knowledge base of the tool

The accuracy of the tool’s diagnostic suggestions was evaluated at each of the different time points of the hospital stay. The list of the 5 most probable conditions provided by the tool was recorded for visit 1, visit 2, and visit 2.1. For the same time points, a maximum of 5 diagnoses were provided in the medical record. If <5 diagnoses were provided in the hospital, the total number of condition suggestions for the case from the DDSS was reduced accordingly. The first listed diagnosis in the ED and the final discharge diagnosis from the hospital were defined as the gold-standard diagnosis for visits 1, 2, and 2.1. The top 1 diagnosis, the top 3 diagnoses, and the top 5 diagnoses provided by the DDSS disease *probability* list were compared with this gold-standard main diagnosis for the 3 different visits. Furthermore, the proportion of diagnoses included in the knowledge base of the tool was assessed. In addition, the missing potentially important information of the findings and symptoms for each case was analyzed and categorized.

### Matching of Diagnoses

The first 5 diagnoses from the DDSS and the first 5 diagnoses from the medical record for each visit were shown to 3 different physicians separately and independently following the completed data collection. They decided whether the diagnosis from the DDSS matched the diagnosis from the treating physician at the different time stamps. This process was necessary as the naming and, therefore, the interpretation of the matching of the diagnoses were not standardized. The 3 physicians did not see the entire case, only the diagnosis lists. They had different levels of clinical expertise and knowledge of the DDSS and the patient case. None of the physicians were involved in anamnesis, clinical examination, or treatment of the patient. The three physicians comprised (1) the study physician, who was involved in the data collection and entry into the DDSS prototype and was therefore familiar with the patient case and who saw the patient in the ED to obtain informed consent and evaluate the appropriateness of the patient for study inclusion; (2) a second independent physician who was an experienced senior physician and fellow of internal medicine and cardiology; and (3) a third physician with several years of work experience in the Medical Knowledge Team of Ada Health GmbH in Berlin and detailed knowledge of the available medical content and the reasoning engine of the DDSS and who also saw the DDSS case in detail to analyze potential user dependencies.

Precise matching criteria were not specified; instead, the physicians were directed to use their experience to decide on the appropriateness of the DDSS condition suggestions. The reasons for inaccurate suggestions of diagnoses were analyzed and categorized. Several causes per case could be assigned, and the related proportions were calculated.

### Statistical Analysis

The top-1, top-3, and top-5 performance of the Ada DDSS prototype condition suggestion for each of the visits, with comparison against the ratings by the 3 physicians, were compared using descriptive statistics and tests appropriate for categorical data. Chi-square tests were used to test whether the proportion of correct answers was drawn from the same distribution, with the application of this test across all visits, once for each of the metrics for comparison (top 1, top 3, and top 5 matching condition suggestions) for each of the 3 physicians’ ratings, to be followed in case of a significant difference by post hoc 2-sided pairwise Fisher exact tests [16]. *P* values were corrected for multiple comparisons using the Benjamini–Hochberg procedure [17], guided by the interpretation of Armstrong [18], and considered significant if <.05.

### Data Processing and Ethical Approval

The conducted study complied with the ethical principles of the World Medical Association Declaration of Helsinki [19]. Ethical approval was obtained from the Ethics Commission Nord-West-Schweiz (reference 2020-00095, date of approval January 24, 2020). All data were stored and transferred in a pseudonymized form. Data processing and transfer were performed in accordance with national and local guidelines. An order data processing agreement was made between the University Hospital Basel and Ada Health GmbH.

## Results

### Patient Characteristics

A total of 33 patients with dyspnea were considered for inclusion, of which 61% (20/33) cases were included and 33% (11/33) were excluded because of direct discharge from the ED without referral to the ward. The refusal rate was low (2/33, 6%). The resulting study population consisted of 40% (8/20) women and 60% (12/20) men aged 54 to 93 years (mean 74 years, SD 10.44 years).

### Feasibility Measures

To create a case in the DDSS prototype, it is required to enter basically all recorded patient data, which is time-consuming, especially in a setting as time-limited as the ED. The data entry took 1.5-2 hours per patient. The checklist used in the ED by the study physician resulted from a pilot round that preceded this study and was a key component of the initial feasibility findings. It was accepted at the outset of the study that the novel user interface of the DDSS prototype was not yet fully developed for use in parallel to every patient’s examination or consultation. The checklist, observational recording, and non–real-time use of the system allowed for the identification of how such a prototype DDSS would need to be involved in capturing the high speed and complexity of clinical data delivery.

### Usability

#### Overview

The Ada DDSS could be usable in the research setting; however, the research team considered that it required optimization before it could be adopted in everyday use in the ED.

Usability insights from our study were principally related to the DDSS *main page* (ie, the *Case analysis* page). This interface consists of three sections: findings and symptoms on the left, case dashboard in the middle, and diagnosis suggestions on the right side (Figure 1).

#### Usability of the Findings Section

The findings were easily located in the search function via several synonyms and terms by the study physician. However, the search engine contained some subcategories and finding synonyms that were sometimes misleading. For more specific findings such as *orthopnea*, the superordinate category *dyspnea* must first be selected and provided with corresponding attributes (in this case, *occurs while lying flat*). This led to a time-consuming search for the right designations by the study physician.

During case input in this study, the finding suggestions were rarely used as the ranking by probability often did not match the physician’s natural clinical workflow.

#### Dashboard Usability

If a finding was added via search function or the list of relevant suggestions (left panel in Figure 1), it must be declared as *present* or *absent* before it was transferred to the case dashboard. Once added to the case, it was not immediately recognizable to the physician how the finding had been marked, which confused the study physician during his work. The color of the contribution line indicates the presence or absence of the finding, but the finding itself is not marked in either way.

The clarity of the presentation of the symptom constellation in relation to the diagnosis list by the contribution lines creates transparency for the user on how the reasoning engine is working. This is one of the main advantages of this DDSS prototype in comparison with others according to the study team.

#### Usability of the Diagnostic Suggestions

As soon as the first finding was entered, the 5 most probable suggested diagnoses were automatically transferred to the case dashboard when switching from the consultation page to the main page.

Once they were listed on the case dashboard, the diagnoses did not update themselves automatically when adding or deleting information. The *probability* and *fit* lists of potential diagnoses on the right side, in contrast, changed in real time, which was confusing for the study personnel.

### Accuracy of Suggested Diagnoses

#### Overview

The results for the accuracy of the DDSS suggested diagnoses are shown in Table 1.

**Table 1:** Accuracy of suggested diagnoses compared with the gold-standard diagnosis (N=20).

	M1^a^				M2^b^				M3^c^		
	(%)	(n/N)	(SD)	(%)		(n/N)	(SD)	(%)		(n/N)	(SD)
Visit 1	18	3.67/20	0.58	42		8.33/20	1.53	57		11.33/20	0.58
Visit 2	35	7/20	1.00	62		12.33/20	1.53	75		15/20	1.00
Visit 2.1	40	8/20	1.00	62		12.33/20	1.53	80		16/20	1.00

^a^First diagnosis listed.

^b^One of the top 3 diagnoses listed.

^c^One of the top 5 diagnoses listed.

The table shows the average top-1, top-3, and top-5 accuracy of the DDSS’s suggestions compared with the gold-standard diagnosis at the different visits, with assessment of the matching of the diagnosis suggestions by physicians with different levels of clinical and tool experience.

Different reasons for incorrect suggestion at the time of diagnosis could be identified and are listed in the following sections.

#### Multimorbidity or Multiple Confirmed Diagnoses or Symptom as Diagnosis

Inpatients in the department of internal medicine often have >1 diagnosed disease, either as known pre-existing diseases or as unknown diseases diagnosed during the inpatient stay. The DDSS prototype seems to focus its reasoning on the evaluation of 1 main diagnosis and, thus, multimorbidity seems to be one of the biggest challenges in correcting condition suggestions. In a few cases, this was the main reason for an incorrect diagnostic suggestion in the DDSS.

In half of the cases (10/20, 50%), the treating physician did not provide a working or final diagnosis compliant with the International Classification of Diseases, 10th revision, as the first listed diagnosis but instead provided a list of several potential working diagnoses or a presenting complaint, which made assessment of the accuracy of the DDSS suggestion impossible. This was especially true for visit 1, when the patient was transferred to the ward for further investigation.

#### Missing Entities in DDSS Knowledge Base

Another aspect that led to incorrect condition suggestions was the lack of relevant entities in the knowledge base of the tool, which limited its ability to suggest a diagnosis. In all these cases, there were one or more relevant diagnostic findings missing (Table 2). In addition, in 20% (4/20) of the cases, the final diagnosis did not exist in the DDSS knowledge base.

**Table 2:** Coverage of symptoms and findings in the medical knowledge base of the diagnostic decision support system (N=20).

Coverage category	Cases with missing relevant information, n (%)
Diagnostic findings (including laboratory, imaging, and histology)	20 (100)
Medical history (pre-existing condition, social, family, and medication)	20 (100)
History or examination findings	9 (45)
Attributes (investigation findings and factors)	6 (30)
Final diagnosis (first diagnosis)	4 (20)
Physiological finding≠negative pathological finding	4 (20)

#### User Input Dependencies

In a number of cases, the level of user experience with the tool was a decisive criterion for the subsequent accuracy of the diagnostic suggestions. In 10% (2/20) of the cases, it was essential for the physician inputting information into the DDSS to know the precise finding name in the DDSS to enable the system to provide accurate diagnosis suggestions.

### Coverage of Diagnoses in the DDSS

For the analysis of the coverage of the clinical diagnoses in the knowledge base of the DDSS prototype (Table 3), a maximum of 5 confirmed diagnoses was considered. There was a total of 186 diagnoses for all cases. Each exact match was considered. In addition, each disease in the differential diagnosis list provided by the hospital was calculated as 0.5 if the diagnosis existed in the DDSS but not in the exact specification, grade, or localization described by the treating physician.

**Table 3:** Coverage of diagnoses in the knowledge base of the diagnostic decision support system.

Item	Visit 1	Visit 2
Sum of diagnoses	93	93
Sum of matches	56.5	65.5
Proportion (%; matches/diagnoses)	61	70

In all the cases, there were one or several entities (out of a large number of relevant entities for each case) that could not be found via the search function in the DDSS (Table 1). Those that could not be entered were mainly diagnostic findings, such as radiologic, laboratory, and histologic findings. In addition, the DDSS did not provide the possibility to report the medical history of pre-existing conditions, medications, or social and familial anamnesis.

## Discussion

### Principal Findings

Regarding the functionality and usability of the tool, it can be summarized that the dynamically interactive DDSS has high potential, with limitations. It showed convincing performance in its clarity of presentation (including transparency of the working of the medical engine) and provided a user-friendly interface. However, the tool as currently developed is not perfectly suited to acute medical settings such as the ED as manual case entry is very time-consuming.

The findings on the DDSS disease suggestion accuracy indicate that it could provide accurate results in the clinical inpatient setting for the many patients who had dyspnea as the main presenting complaint. The symptom analysis algorithm of the DDSS seems to weigh the order of the symptoms present in a case, the likelihood of a finding for a diagnosis, and the epidemiology. Unlikely symptoms, absent common symptoms, and misleading findings as well as an atypical age of the patient for a disease or an uncommon primary anatomical site of involvement might lead to misdiagnosis in the system. These results should be interpreted with caution at this stage as the study setting was observational, and real-world interventional studies are suggested for confirmation.

Another finding of this study is that, although the medical professional knowledge base already covers many different findings, it is nonetheless incomplete in some areas. Many findings from investigative procedures in the hospital are not yet provided by the tool and, in some cases, this decreased the accuracy of the suggested diagnoses.

The diagnosis suggestions also depend to a large degree on appropriate user input. The treating physician’s medical knowledge and skills as well as the expertise of the study team with the use of the tool could potentially have influenced the outcome of this study. A higher experience in all of these fields might improve the accuracy outcome and should be investigated separately. It is acknowledged that the use of a DDSS of this type in a real-world setting requires training of personnel on the use of the system and how to obtain the best results from the tool.

The results from this study suggest that the Ada DDSS could have the potential to support the clinician in their daily work, but an enlargement of its professional medical knowledge base and a larger-scale evaluation study would be necessary beforehand.

### Possible Improvements to the Ada DDSS

This feasibility study found some areas where the DDSS could be improved. The search and selection of symptoms and findings is one of the areas with the greatest potential for improvement. A structure that follows the logic of how physicians think (eg, a step-by-step selection starting with the examination or the investigation method, ending with the proofing pathological finding, and dividing the findings into categories) could improve the intuitive usability for physicians. The manner of displaying the highest–information-gain symptoms and finding suggestions on the case dashboard could thereby also be improved. In its current stage, this list shows a collection of unsorted and uncategorized symptoms and findings generated by the reasoning engine.

As it is up to the using physician to select the relevant diagnoses from the diagnosis lists and add them to the case dashboard, this should be made clear to the user, or the list of added diagnoses should automatically update itself. It would be helpful to signal to the user at a glance whether the finding was marked as absent either by using a different font color or by placing a cross in front of the finding. This would be a simple change with a large impact on usability.

The routine adoption of the tool in a highly dynamic setting such as the ED could only be achieved after a reduction in active effort to enter information. Automatic integration of basic patient and anamnestic information as well as further extraction of information from the electronic medical record could save a large amount of time for the treating physician and decrease potential bias because of user dependencies. The acceleration of rare disease diagnosis [12] and the higher accuracy in the inpatient setting also indicate that, in its current form, the DDSS is more suited to those disciplines. Used in the correct medical setting, it has the potential to support the physician in their decision-making process by showing new pathways of diagnostic reasoning and suggesting unintentionally ignored diagnoses. The pooling of the immense medical knowledge available has the potential to extend the medical disease spectrum of a physician in their routine work. If this functionality is extended through a wider professional medical knowledge base, it has the potential to assist in rational test choice and avoid important diagnostic investigations being overlooked.

Many patients in the internal medicine ward have >1 diagnosis. Multiple diagnoses at the time of discharge from the ED to the ward or from the ward to an outpatient setting led to a lower accuracy in the tool. This seems to be one of the biggest challenges and should be a focus area for the improvement of the DDSS.

Currently, medication and information about the therapy of a patient cannot be entered into the DDSS. This results in the underconsideration of possible therapeutic symptom improvements, therapy failure, or medicinal side effects in the probability estimation of the diagnosis suggestions.

Social or family history has a rudimentary representation in the DDSS and, consequently, follow-up or secondary diseases, exacerbation of an existing disease, social measures, or familial predisposition are underreflected.

An optimized and extended Ada DDSS based on the system evaluated here could save time and improve investigative and diagnostic efficiency and quality, thereby improving health economic outcomes [20,21]. These effects need to be assessed in future studies.

### Strengths and Limitations

This study has several strengths and limitations. The study design, with a real-world setting, prospective data collection, and the shadowing of the treating physician by a study physician without any interference with usual care, as well as the measurement of accuracy analysis through the use of a panel of 3 physicians with different clinical backgrounds, was important for the study strengths.

The small number of cases is a limitation of the study, as is the focus on only 1 main presenting symptom for the selection of participants. Both factors offer potential for selection bias; however, they are appropriate for this stage of feasibility evaluation.

Our study showed that the DDSS condition suggestions are user-dependent—the level of knowledge, expertise, and familiarity had a large impact on accuracy. In some cases, an additional finding, which was difficult to find via the tool’s search function because of specific wording, led to a completely altered differential diagnosis list and accuracy. As the case set was small and a large range of physician users was not explored, the range of user dependency of the DDSS was not precisely quantified. The focus of this study was to assess the feasibility and usability of the novel interface of the DDSS. It was not intended as a validation of the accuracy of its diagnosis suggestions. A future large-scale study with blinded, maybe automated data entry after consistent training of the clinical users of the system should be performed to evaluate and validate the accuracy of a ready-for-market DDSS.

### Conclusions

This study provides insights into the applicability and performance of the DDSS prototype and the potential of the highly dynamic case input interface for medical professionals, especially in an inpatient setting. The clear and user-friendly presentation of a clinical patient case, with a transparent visual explanation of the algorithmic decisions, is the outstanding novelty of the tool used in this study.

At its current stage of development, the DDSS prototype has some limitations regarding the automation of data input, the accuracy of the diagnostic suggestions, and the completeness of the integrated medical knowledge. Data entry and analysis are still highly user-dependent; however, this could be minimized through training and experience.

The results of this study provide a basis for the further development of this and related tools. Further development of dynamic and highly transparent DDSS case interfaces is warranted and, once systems are optimized further, setting-appropriate studies are required to evaluate clinical outcomes.

## Abbreviations

DDSS: diagnostic decision support system
ED: emergency department
